# Synthesis, Anti-microbial and Molecular Docking Studies of Quinazolin-4(3H)-one Derivatives

**DOI:** 10.3390/molecules19078725

**Published:** 2014-06-25

**Authors:** Yahia Nasser Mabkhot, Munirah S. Al-Har, Assem Barakat, Fahad D. Aldawsari, Ali Aldalbahi, Zaheer Ul-Haq

**Affiliations:** 1 Department of Chemistry, College of Science, King Saud University, P.O. Box 2455, Riyadh 11451, Saudi Arabia; E-Mails: mun_eera2010@hotmail.com (M.S.A.-H.); faldossri@kacst.edu.sa (F.D.A.); aaldalbahi@ksu.edu.sa (A.A.); 2 Department of Chemistry, College of Sciences, Hail University, P.O. Box 2440, Hail 81451, Saudi Arabia; 3 Department of Chemistry, Faculty of Science, Alexandria University, P.O. Box 426, Ibrahimia 21321, Alexandria, Egypt; 4 King Abdulaziz City for Science and Technology, P.O. Box 6086, Riyadh-11442, Saudi Arabia; 5 Dr. Panjwani Center for Molecular Medicine and Drug Research, International Center for Chemical and Biological Sciences, University of Karachi, Karachi-75210, Pakistan; E-Mail: zaheer.qasmi@iccs.edu

**Keywords:** quinazolinone, antimicrobial agents, streptomycin, clotrimazole, molecular docking

## Abstract

In this work, synthesis, antimicrobial activities and molecular docking studies of some new series of substituted quinazolinone **2a**–**h** and **3a**–**d** were described. Starting form 2-aminobenzamide derivatives **1**, a new series of quinazolinone derivatives has been synthesized, in high yields, assisted by microwave and classical methods. Some of these substituted quinazolinones were tested for their antimicrobial activity against Gram-negative bacteria (*Pseudomonas aeruginosa* and *Esherichia coli*) and Gram-positive bacteria (*Staphylococcus aureus*, and *Bacillus subtilis*), and anti-fungal activity against (*Aspergillus fumigatus*, *Saccharomyces cervevisiae*, and *Candida albicans*) using agar well diffusion method. Among the prepared products, 3-benzyl-2-(4-chlorophenyl)quinazolin-4(3H)-one (**3a**) was found to exhibits the most potent *in vitro* anti-microbial activity with MICs of 25.6 ± 0.5, 24.3 ± 0.4, 30.1 ± 0.6, and 25.1 ± 0.5 µg/mL against *Staphylococcus aureus*, *Bacillus subtilis*, *Pseudomonas aeruginosa* and *Esherichia coli*, respectively. Compound **3a** was found to exhibits the most potent *in vitro* anti-fungal activity with MICs of 18.3 ± 0.6, 23.1 ± 0.4, and 26.1 ± 0. 5 µg/mL against *Aspergillus fumigatus*, *Saccharomyces cervevisiae*, and *Candidaal* *bicans*, respectively.

## 1. Introduction

In the last decade, quinazolines have been extensively studied in medicinal chemistry. The quinazolinone scaffold is considered to be a motif structure. Quinazolinone ring system is found in a variety of bioactive natural as well as synthetic products. Many natural products contain quinazolinone core structures for example asperlicin C, sclerotigenin, circumdatin F, benzomalvin A, and many others have been documented as biologically important molecules [1,2,3]. Some synthetic quinazolinones, such as ispinesib, raltitrexed, halofuginone, tempostatin, *etc.* have been in the market or are currently in clinical trials for various cancer treatments. Quinazolines have exhibited therapeutic activities including antibacterial [4,5], antiviral [6], antifungal [7,8], antimalarial [9], antihypertensive [10], anticancer [11,12,13], diuretic [14,15], inhibition of derived growth factor receptor phosphorylation [16], antagonism of ghrelin receptor [17], anticonvulsant [18], COX-2 inhibitory activities [19,20] analgesic and anti-inflammatory.

Particularly, enzyme Sortase A involves in the pathogenesis of variety of bacterial infections, including respiratory tract, bloodstream, skin and tissue infection which serve to anchor some proteins responsible for virulence mainly by Gram +ve bacteria. Sortase A has been attracted great interest as potential drug targets since decades [21]. The inhibition of *Sortase A* activity results in the separation of *S. aureus* from the host cells and ultimately alleviation of the infection. We used these newly synthesized active inhibitors to explore the binding cavity of *Staphylococcus* aureus Sortase A using GOLD docking program [21].

Recently, Mabkhot and co-workers have been involved in a research program aimed to development of new synthetic strategies for novel bioactive molecules and evaluation of their biological activity [22,23,24,25,26,27,28].

In this paper, we synthesized some new substituted quinazolinones as potential anti-microbial agents starting form 2-aminobenzamdie derivatives. The validity of this hypothesis was confirmed through preliminary *in vitro* anti-bacterial and anti-fungal screening of the desired molecules and their molecular docking studies.

## 2. Results and Discussion

### 2.1. Chemistry

**Scheme 1:**
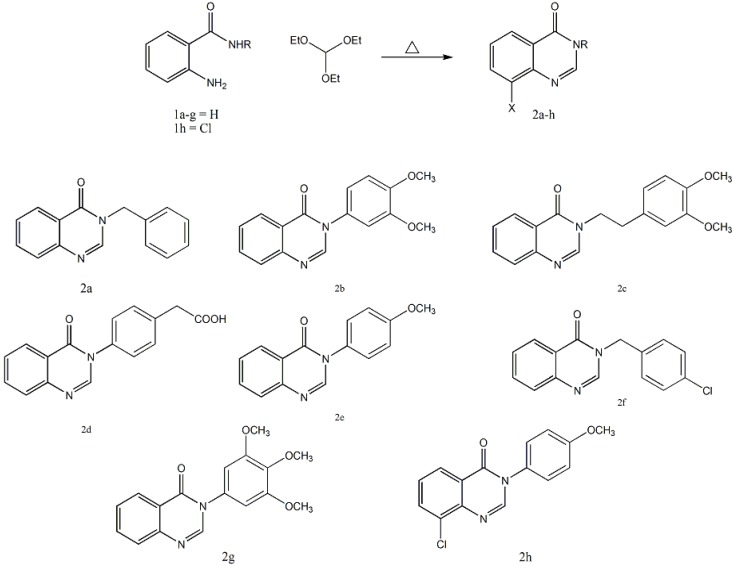
Synthesis of quinazolin-4(3H)-one derivatives **2a**–**h** from 2-aminobenzamide derivatives **1a**–**h**.

Synthesis of the desired compounds **2a**–**h** was achieved by allowing 2-aminobenzamide derivatives **1a**–**h** [29,30] to undergo ring closure either with triethyl orthoformate under classical reflux condition as shown in Scheme 1. Alternatively, **2a**–**f**, **h** can be obtained by microwave mediated methodology. It is proposed that the product **2a**–**h** was formed via initial nucleophilic addition of amide group into electrophilic carbon followed ring closure and elimination of three molecules of EtOH to give the desired products **2a**–**h**. Assignment of structures of **2a**–**h** is based on elemental analysis and spectral data. Their IR spectra showed the disappearance of the characteristic absorption bands of NH & NH_2_ groups. Their ^1^H-NMR spectra showed in each case a characteristic singlet assigned to proton of N=CH. Their MS spectra are matched with the designed structures.

**Scheme 2:**
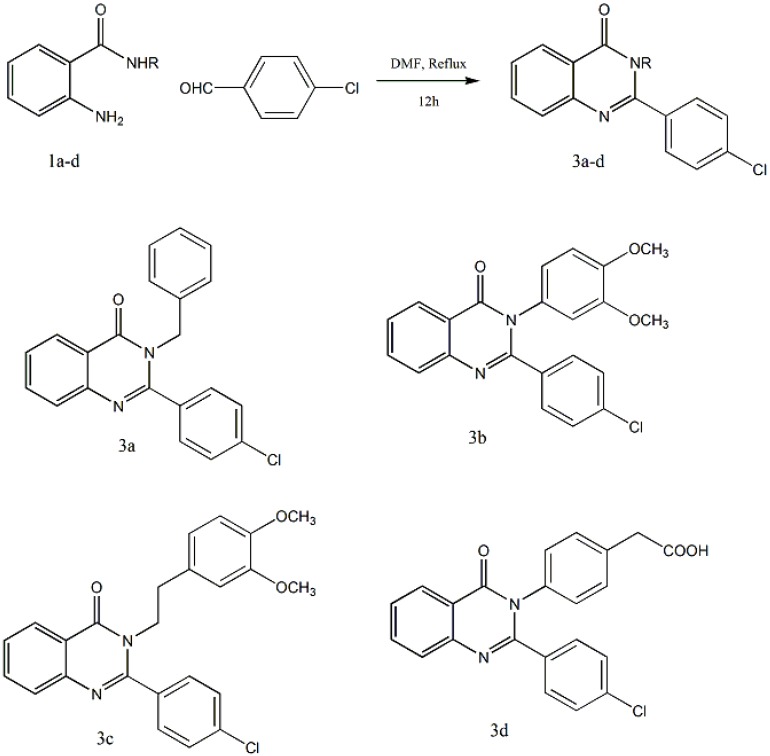
Synthesis of quinazolin-4(3H)-one derivatives **3a**–**d** from 2-aminobenzamide derivatives **1a**–**d**.

Next, compounds **3a**–**d** were synthesized by reaction of 2-aminobenzamide derivatives **1a**–**d** with *p*-chlorobenzaldehyde, in DMF under reflux for 12 h, as shown in Scheme 2. On the other hand, microwave mediated methodology gave the same **3a**,**b**. It is assumed that the products **3a**–**d** were formed via initial nucleophilic addition of amide to carbonyl group followed ring closure and elimination of H_2_O to give the desired products **3a**–**d**. Elucidations of the chemical structures of **3a**–**d** are inferred from their spectroscopic and analytical data. Their IR spectra showed the disappearance of the characteristic absorption bands of NH & NH_2_ groups.The NMR and MS spectra are matched with the designed structures.

### 2.2. Antimicrobial Evaluation

Antimicrobial activity of the synthesized compounds has been screened against micro-organisms representing Gram-(+ve) bacteria (*Staphylococcus aureus* and *Bacillus subtilis*), Gram-(−ve) bacteria (*Escherichia coli* and *Pseudomonas aeruginosa*), and fungi (*Aspergillus fumigatus*, *Saccharomyces cerevisiae* and *Candida albicans*), using the bioassay technique of antibiotics as antibacterial and antifungal standard drug specified in the US pharmacopeia at 25 µg/mL. Among the screened compounds, only compound **3a** showed a potent inhibitory effect against Gram-(+ve) bacteria (*Staphylococcus aureus* and *Bacillus subtilis*) and Gram-(−ve) bacteria (*Escherichia coli* and *Pseudomonas* *aeruginosa*). Compound **3a** found to be as similar potency as the standard drug (streptomycin). Nevertheless, **3a** showed the strong inhibitory effect against fungi (*Saccharomyces Cerevisiae*, *Aspergillus fumigatus* and *Candida albicans*). The results indicate that compounds **3a** has excellent biological activity and can be subjected for further evaluation for example enzyme inhibition. Clotrimazole and Streptomycin were used as standards [31]. The results obtained are listed in Table 1.

**Table 1:** Antimicrobial evaluation of the synthesized molecules.

Comp. No.	Gram-Postive Bacteria		Gram-Negative Bacteria		Fungi		
	*Staphylococcus aureus*	*Bacillus subtilis*	*Pseudomonas aeruginosa*	*Escherichia coli*	*Aspergillus fumigatus*	*Saccharomyces cerevisiae*	*Candida albicans*
**2b**	7.8 ± 0.4	9.2 ± 0.3	9.7 ± 0.3	8.9 ± 0.3	9.5 ± 0.4	10.3 ± 0.4	10.5 ± 0.4
**2c**	12.3 ± 0.3	13.1 ± 0.4	18.4 ± 0.5	15.2 ± 0.4	9.4 ± 0.3	15.2 ± 0.4	13.3 ± 0.4
**2d**	9.8 ± 0.3	12.2 ± 0.2	14.1 ± 0.4	13.8 ± 0.3	12.1 ± 0.4	13.7 ± 0.3	12.8 ± 0.2
**2g**	10.3 ± 0.2	11.1 ± 0.4	10.9 ± 0.4	9.8 ± 0.3	11.3 ± 0.5	12.2 ± 0.4	11.4 ± 0.3
**2h**	9.1 ± 0.4	9.6 ± 0.3	9.6 ± 0.3	9.8 ± 0.3	10.4 ± 0.4	11.3 ± 0.4	12.2 ± 0.4
**3a**	25.6 ± 0.5	24.3 ± 0.4	30.1 ± 0.6	25.1 ± 0.5	18.3 ± 0.6	23.1 ± 0.4	26.1 ± 0.5
**3b**	10.4 ± 0.3	11.3 ± 0.3	11.1 ± 0.2	10.8 ± 0.3	9.8 ± 0.2	10.8 ± 0.4	10.3 ± 0.5
**clotrimazole**					18.3 ± 0.6	23.1 ± 0.4	26.1 ± 0.5
**streptomycin**	25.6 ± 0.5	24.3 ± 0.4	30.1 ± 0.6	25.1 ± 0.5			

Inhibition zones (mm).

### 2.3. Molecular Docking Studies

**Figure 1:**
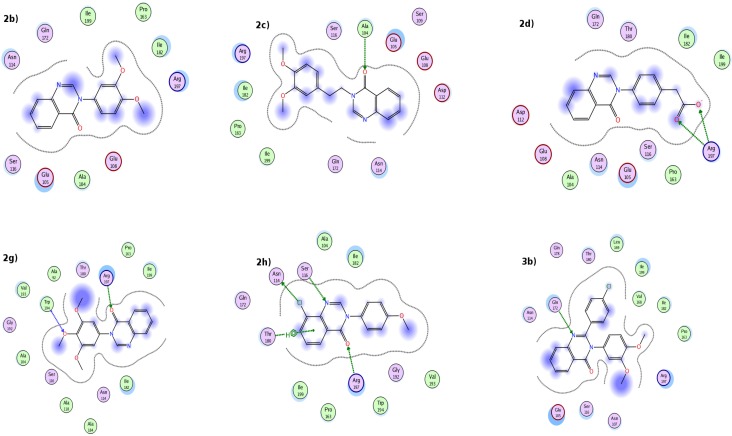
Docked ligand at the receptor binding site. This picture represent 2D-interactions for the newly synthesized inhibitors within the binding pocket of target receptor using Poseview.

The antimicrobial potency of all the newly synthesized compounds were subjected for further docking studies to explore the binding pattern against methicillin resistant *Staphylcoccus aureus* (*MRSA*) [21]. PDB (ID 1T2W) [32] with 1.80 Å resolution was retrieved from Brookhaven Protein Data Bank. From our previous studies residues including Ala92, Ala104, Glu105, Ala118, Pro163, Leu169, Gln172, Thr180, Ile182, Trp194 and Ile199 were found to be in the active site of the receptor, responsible for hydrophobic interactions. While Arg197 was the hotspot residue showing significant hydrogen bond within the binding pocket [21]. Conformational search of ligands were investigated *via* Gold docking program with extensive genetic algorithm. In this study, ten conformers were generated for each ligand using default parameters. Docking of all the newly synthesized inhibitors showed hydrogen bond interactions with Arg197 mentioned in Figure 1, supported our by previous findings [21].

**Figure 2:**
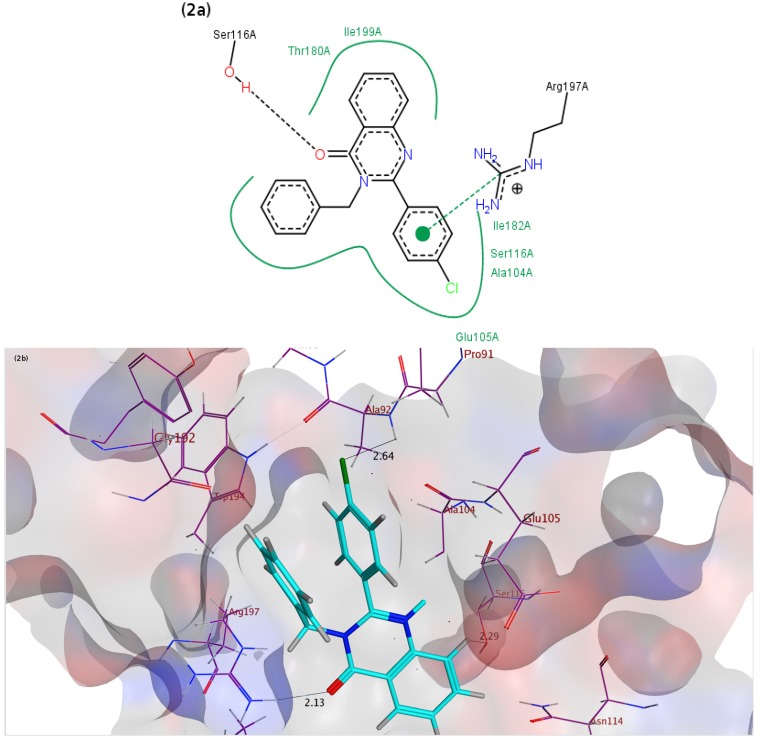
Molecular Docking Interaction diagram for the most potent compound **3a**. Panel (**a**) is a two-dimensional representation of the docked pose by Poseview. Panel (**b**) representing three-dimensional view by MOE.

The most active newly synthesized compound as an antimicrobial agent **3a** showed potent inhibitory activity; involve in hydrogen bonding interaction with oxygen of quinazoline moiety at 2.13 Å with Arg197. While the hydroxyl group of Ser116 showed strong hydrogen bond interaction with Ser116 at a bond distance of 2.29 Å and chloro moiety of benzene ring attached to quinazoline showed hydrogen bond with Ala92 with a distance of 2.64 Å. Hydrophobic interaction also observed between Trp194 and first benzene ring of the quinazoline moiety (Figure 2).

## 3. Experimental Section

General: The ^1^H-NMR and ^13^C-NMR spectra were run in DMSO-*d*_6_ and recorded on a Varian Mercury or Jeol-400 NMR spectrometer. Coupling constants *J* are given in Hz and chemical shifts (δ) are referred in ppm and related to that of the solvent. Abbreviations for multiplicity are as follows: m (multiplet), q (quadruplet), t (triplet), d (doublet) and s (singlet). IR spectra were recorded on a Perkin Elmer FT 1000 spectrophotometer using KBr pellets. Mass spectroscopy was measured on a Shimadzu GCMS-QP 1000 EX mass spectrometer at 70 eV. Melting points were measured on a Gallenkamp melting point apparatus in open glass capillaries and are uncorrected. Elemental analysis was carried out on an Elementar Vario EL analyzer.

### 3.1. General Procedure for the Preparation of Qquinazolin-4(3H)-one Derivatives **2a**–**h**

**Method** **A** (**2a**–**h**): To a mixture of 2-aminobenzamide derivatives **1a**–**h** (2 mmol) was added triethyl orthoformate (2–3 mL) and the reaction mixture was heated under reflux for 12 h. The reaction was monitored by TLC (EtOH: CHCl_3_), then left to cool to RT. The precipitated solid was filtered off, washed with diethyl ether and recrystallized from the proper solvent to afford the corresponding product **2a**–**h**.

**Method** **B** (**2a**–**f**, **h**): A mixture of 2-aminobenzamide derivatives **1a**–**g**, **h** (2 mmol) and triethyl orthoformate (5–10 mmol) in the presence of view drops of DMF, was exposed to microwave irradiation (140–420 w) for 4–10 min. The reaction mixture was left to cool to RT and methanol was added. The formed solid product was filtered off, washed with diethyl ether and recrystallized from the appropriate solvent to afford the corresponding product **2a**–**g**, **h**.

*3-Benzylquinazolin-4(3H)-one* (**2a**). This compound was prepared from 2-amino-*N*-benzylbenzamide **1a** according to method A or method B (4 min, 280 W), and recyrtsallized from ethanol afford **2a** as shining white needles; yield (82**^a^**, 65**^b^**%); m.p. 116 °C; IR *ν*_max_ (KBr) 1630.75, 1578.88 cm^−1^; ^1^H-NMR (400 MHz, DMSO-*d*_6_) (ppm): *δ* 8.58 (1H, s), 8.16 (1H, d, *J* = 8.0 Hz), 7.84 (1H, t, *J* = 8.0 Hz), 7.70 (1H, d, *J* = 8.0 Hz), 7.55 (1H, t, *J* = 8.0 Hz), 7.28–7.38 (5H, m), 5.20 (2H, s, CH_2_); ^13^C-NMR: *δ* 50.0, 122.20, 126.68, 127.77, 127.83, 128.21(2C), 128.24, 129.21(2C), 135.00, 137.42, 148.48, 148.58, 160.7; MS *m*/*z* (%): 236[M^+^](C_15_H_12_N_2_O); Anal. For C_15_H_12_N_2_O (236.27) calcd; C, 76.25; H, 5.12; N, 11.86; Found: C, 76.24; H, 5.12; N, 11.86.

*3-(3,4-Dimethoxyphenyl)quinazolin-4(3H)-one* (**2b**). Prepared from 2-amino-*N*-(3,4-dimethoxyphenyl)benzamide (**1b**) according to method A or method B (6 min, 280 W), and recyrstallized from ethanol to afford **2b**, dark powder; yield (99**^a^**, 70**^b^**%); m.p. 140 °C; IR *ν*_max_ (KBr) 1669.14, 1595.03 cm^−1^; ^1^H-NMR (400 MHz, DMSO-*d*_6_) (ppm): *δ* 8.31(1H, s), 8.20 (1H, d, *J* = 8.0 Hz), 7.88 (1H, t, *J* = 8.0 Hz), 7.74 (1H, d, *J* = 8.0 Hz), 7.60 (1H, t, *J* = 8.0 Hz), 7.18 (1H, s), 7.1 (1H, d, *J* = 8.0 Hz), 7.06 (1H, d, *J* = 8.0 Hz), 3.77,3.83 (each 3H, s, 2 (OCH_3_)); ^13^C-NMR: *δ* 58.0, 112.13 (2C), 120.11, 122.51, 126.98, 127.84, 127.91, 130.96, 135.14, 148.13, 148.31, 149.39, 149.49, 160.7; MS *m*/*z* (%): 282[M^+^](C_16_H_14_N_2_O_3_) (26.56), 222 (69.23), 193 (51.56), 193 (50.77), 43 (80.00), 55 (100); Anal. for C_16_H_14_N_2_O_3_ (282.29) calcd; C, 68.07; H, 5.00; N, 9.92; Found: C, 68.10; H, 5.03; N, 9.91.

*3-(3,4-Dimethoxyphenethyl)quinazolin-4(3H)-one* (**2c**). Prepared from 2-amino-*N*-(3,4-dimethoxyphenethyl)benzamide **1c** according to method A, as beige powder; yield (95**^a^**%); m.p. 140 °C; IR *ν*_max_ (KBr) 1681.65, 1642.74 cm^−1^; ^1^H-NMR (400 MHz, DMSO-*d*_6_) (ppm): *δ* 8.76 (1H, s), 8.36 (1H, d, *J* = 7.8 Hz), 7.65 (1H, d, *J* = 7.8 Hz), 7.46 (1H, t, *J* = 7.8 Hz), 7.12 (1H, t, *J* = 7.8 Hz), 6.83–6.87 (2H, m), 6.75 (1H, d, *J* = 8.0 Hz), 3.71 (6H, s, (2(OCH_3_)), 3.47 (2H, t, CH_2_), 2.79 (2H, t, CH_2_); ^13^C-NMR: *δ* 25.3, 34.9, 55.9, 56.0,112.37, 113.07, 120.93, 121.09 (2C), 121.65, 123.00, 128.47, 132.24, 132.32, 139.36, 147.78, 149.10, 168,7; MS *m*/*z* (%): 310[M+](C_18_H_18_N_2_O_3_) (15.62), 293 (100), 275 (35.94), 28 (21.87), 44 (20.31), 69 (18.75); Anal. For C_18_H_18_N_2_O_3_ (310.35) calcd; C, 69.66; H, 5.85; N, 9.03; Found: C, 69.64; H, 5.83; N, 9.05.

*2-(4-(4-Oxoquinazolin-3(4H)-yl)phenyl)acetic Acid* (**2d**). **Compound** **2d** was prepared from 2-(4-(2-aminobenzamido)phenyl)acetic acid **1d** according to method A or method B (5 min, 280 W), and recrystallized from ethanol afford **2d** as, brownish powder; yield (85**^a^**, 70**^b^**%); m.p. 267 °C; IR *ν*_max_ (KBr) 3678.56, 3449.51, 1687.32, 1611.24, 1564.26 cm^−1^; ^1^H-NMR (400 MHz, DMSO-*d*_6_) (ppm): *δ* 8.35 (1H, s), 8.21 (1H, d, *J* = 7.7 Hz), 7.89 (1H, t, *J* = 7.7 Hz), 7.75 (1H, d, *J* = 7.7 Hz), 7.60 (1H, t, *J* = 7.7 Hz), 7.49 (2H, d, *J* = 8.0 Hz), 7.45 (2H, d, *J* = 8.0 Hz), 3.69 (2H, s, CH_2_); ^13^C-NMR: *δ* 25.3, 122.46, 127.01, 127.79 (2C), 127.89, 127.98, 130.81(2C), 135.23, 136.35, 136.63, 147.78, 148.29, 160.6, 173.1; MS *m*/*z* (%): 280[M^+^](C_16_H_12_N_2_O_3_) (100), 250 (19.05), 235 (92.55), 207 (15.24),129 (32.38), 107 (56.19); Anal. for C_16_H_12_N_2_O_3_ (280.28) calcd; C, 68.56; H, 4.32; N, 9.99; Found: C, 68.55; H, 4.31; N, 10.01.

3-(4-Methoxyphenyl)quinazolin-4(3H)-one (**2e**). **Compound** **2e** was prepared from 2-amino-*N*-(4-methoxyphenyl)benzamide **1e** according to method A or method B (4 min,420 W), and recrystallized from ethanol afford **2e** as shining white needles; yield (99**^a^**, 95**^b^**%); m.p. 195 °C; IR *ν*_max_ (KBr) 1681.54, 1610.01 cm^−1^; ^1^H-NMR (400 MHz, DMSO-*d*_6_) (ppm): *δ* 8.31 (1H, s), 8.20 (1H, d, *J* = 8.0 Hz), 8.20 (1H, d, *J* = 8.0 Hz), 7.88 (1H, t, *J* = 8.0 Hz), 7.74 (1H, d, *J* = 8.0 Hz), 7.60 (1H, t, *J* = 8.0 Hz), 7.46 (2H, d, *J* = 8.0 Hz), 7.10 (2H, d, *J* = 8.0 Hz), 3.83 (3H, s, OCH_3_); ^13^C-NMR: *δ* 56.1, 114.90 (2C), 122.49, 126.98, 127.85, 127.91, 129.24 (2C), 130.86, 135.14, 148.07, 148.34, 159.82, 160.8; MS *m*/*z* (%): 252[M^+^](C_15_H_12_N_2_O_2_) (100), 253 [M^+^+1] (20.80), 254 [M^+^+2] (2.20), 237 (18.30), 209 (10.92), 129 (18.44); Anal. for C_15_H_12_N_2_O_2_ (252.27) calcd; C, 67.82; H, 4.82; N, 12.17. Found: C, 67.81; H, 4.80; N, 12.25.

*3-(4-Chlorobenzyl)quinazolin-4(3H)-one* (**2f**). **Compound** **2f** was prepared from 2-amino-*N*-(4-chlorobenzyl)benzamide **1f** according to method A or method B (2 min, 280 W) and recrystallized from methanol afford **2f** as white powder; yield (88**^a^**, 62**^b^**%); m.p. 155 °C; IR *ν*_max_ (KBr) 1602.24, 1531.10 cm^−1^; ^1^H-NMR (400 MHz, DMSO-*d*_6_) (ppm): *δ* 8.60 (1H, s), 8.15 (1H, d, *J* = 8.0 Hz), 7.83 (1H, t, *J* = 8.0 Hz), 7.70 (1H, d, *J* = 8.0 Hz), 7.31–7.41 (4H, m), 6.56 (1H, t, *J* = 8.0 Hz), 5.19(2H, s, CH_2_); ^13^C-NMR: *δ* 49.9, 126.66, 127.82, 128.74, 150.10, 129.15 (2C), 129.57, 130.23 (2C), 132.40, 132.88, 135.07, 136.40, 148.5; MS *m*/*z* (%): 270[M^+^](C_15_H_11_N_2_OCl); Anal. for C_15_H_11_ClN_2_O (270.71) calcd; C, 66.55; H, 4.10; Cl, 13.10; N, 10.35; Found: C, 66.54; H, 4.11; Cl, 13.09; N, 10.33.

*3-(3,4,5-Trimethoxyphenyl)quinazolin-4(3H)-one* (**2g**). **Compound** **2g** was prepared from 2-amino-*N*-(3,4,5-trimethoxyphenyl)benzamide **1g** according to method A or method B (4 min,280 W), as brownish powder; yield (98**^a^**, 88**^b^**%); m.p. 115 °C; IR *ν*_max_(KBr) 1680.00, 1607.17 cm^−1^; ^1^H-NMR (400 MHz, DMSO-*d*_6_) (ppm): *δ* 8.34 (1H, s), 8.21 (1H, d, *J* = 8.0 Hz), 7.88(1H, t, *J* = 8.0 Hz), 7.75 (1H, d, *J* = 8.0 Hz), 7.60 (1H, t, *J* = 8.0 Hz), 7.17 (1H, s), 6.92 (1H, s), 3.77,3.79 (each 3H, s, 2(OCH_3_)), 3.76 (3H, s, OCH_3_); ^13^C-NMR: *δ* 56.3, 56.7, 106.11 (2C), 122.49, 126.97, 127.84, 127.95, 133.86, 135.20, 138.07, 148.26, 153.09, 153.59, 160.6; MS *m*/*z* (%): 312[M^+^](C_17_H_16_N_2_O_4_) (6.25), 297 (31.25), 194 (100), 165 (25.00), 87 (17.18), 68 (23.44); Anal. for C_17_H_16_N_2_O_4_ (312.32) calcd; C, 65.38; H, 5.16; N, 8.97; Found: C, 65.39; H, 5.15; N, 8.93.

*8-Chloro-3-(4-methoxyphenyl)quinazolin-4(3H)-one* (**2h**). **2h** was prepared from 2-amino-3-chloro-*N*-(4-methoxyphenyl)benzamide **1h** according to method A, or method B(4 min, 280 W) as shining beige powder; yield (97**^a^**, 60**^b^**%); m.p. 175 °C; IR *ν*_max_ (KBr) 1631.68, 1610.34 cm^−1^; ^1^H-NMR (400 MHz, DMSO-*d*_6_) (ppm): *δ* 8.43 (1H,s), 7.57–7.62 (3H, m), 7.43 (1H, d, *J* = 8.0 Hz), 6.92 (2H, d, *J* = 8.8 Hz), 6.66 (1H, t, *J* = 8.0 Hz), 3.74 ( 3H, s, OCH_3_); ^13^C-NMR: *δ* 55.74, 114.26 (2C), 114.94, 115.90, 118.17, 119.48, 122.88 (2C), 128.21, 129.21, 132.42, 145.53, 156.21, 167.30; MS *m*/*z* (%): 28[M^+^](C_15_H_11_N_2_O_2_Cl) (61.50), 288 [M^+^+2] (C_15_H_11_N_2_O_2_Cl) (21.02), 187 (100), 244 (50.94), 43 (39.00), 189 (33.10); Anal. for C_15_H_11_ClN_2_O_2_ (286.71) calcd; C, 62.84; H, 3.87; Cl, 12.37; N, 9.77; Found: C, 62.85; H, 3.87; Cl, 12.36; N, 9.78.

### 3.2. General Method for Preparation of Compounds Derivatives **3a**–**o**

**Method** **A** **(3a**–**d)**: To a solution of 2-aminobenzamide derivatives **1a**–**d** (2 mmol) in DMF (2–3 mL), and *p*-chlorobenzaldehyde (2 mmol) was added. The reaction mixture was heated under reflux for 12 h. The reaction mixture was monitored by TLC (EtOH/CHCl_3_), then left to cool to RT, and poured into cold water (50 mL), the precipitated solid product was filtered off, and dried to afford the corresponding product **3a**–**d**.

**Method** **B** **(3a**,**b)**: A mixture of 2-aminobenzamide derivatives **1a**,**b** (2 mmol) and *p*-chlorobenzaldehyde (2 mmol) in the presence of view drops from DMF, was exposed to microwave irradiation (420–560 w) for 5–10 min. The reaction mixture was left to cool to RT. Water was added and the formed solid product was filtered off afforded the corresponding product **3a**,**b**.

*3-Benzyl-2-(4-chlorophenyl)quinazolin-4(3H)-one* (**3a**). **3a** was prepared from 2-amino-*N*-benzylbenzamide **1a** according to method A, or method B (5 min, 420 W) as white scales; yield (82**^a^**, 63**^b^**%); m.p. 101 °C; IR *ν*_max_ (KBr) 1617.23, 1589.98 cm^−1^; ^1^H-NMR (400 MHz, DMSO-*d*_6_) (ppm): *δ* 7.85 (1H, d, *J* = 8.0 Hz), 7.79 (2H, d, *J* = 8.8 Hz), 7.51–7.56 (3H, m), 7.25–7.36 (7H, m), 4.49 (2H, s, CH_2_); ^13^C-NMR: *δ* 40.3, 119.76, 126.81, 127.45 (2C), 128.02 (2C), 128.89 (2C), 129.52 (2C), 129.98, 131.14 (2C), 132.16, 134.84, 137.12, 139.61, 149.24, 161.74, 166.5; MS *m*/*z* (%): 346 [M^+^] (C_21_H_15_N_2_OCl) (100%), 347 [M^+^+1] (23.00), 348[M^+^+2] (33.61), 349 [M^+^+3] (6.90%), 155 (60.55%), 91 (78.03%); Anal. for C_21_H_15_ClN_2_O (346.81) calcd; C, 72.73; H, 4.36; Cl, 10.22; N, 8.08; Found: C, 72.75; H, 4.36; Cl, 10.23; N, 8.10.

*2-(4-Chlorophenyl)-3-(3,4-dimethoxyphenyl)quinazolin-4(3H)-one* (**3b**). **3b** was prepared from 2-amino-*N*-(3,4-dimethoxyphenyl)benzamide **1b** according to method A, or method B (5 min, 560 W) as shining pale green scales; yield (87**^a^**, 77**^b^**%); m.p. 147 °C; IR *ν*_max_ (KBr) 1615.17, 1561.63 cm^−1^; ^1^H-NMR (400 MHz, DMSO-*d*_6_) (ppm): *δ* 8.04(2H, d, *J* = 8.0 Hz), 7.92 (1H, d, *J* = 8.0 Hz), 7.60–7.65 (3H, m), 7.38–7.41 (3H, m), 7.33 (1H, d, *J* = 8.0 Hz), 6.91 (1H, t, *J* = 8.0 Hz), 3.70, 3.72 (each 3H, s, (2(OCH_3_)); ^13^C-NMR: *δ* 55.8, 56.3, 111.95, 112.61, 119.89, 126.99, 129.60 (2C), 129.77, 130.09, 131.22 (2C), 132.55, 132.89, 134.97, 137.42, 145.62, 149.11,149.20, 162.04, 164.6; MS *m*/*z* (%): 392 [M^+^] (C_22_H_17_N_2_O_3_Cl) (20.90%), 393 [M^+^+1] (5.34), 391[M^+^−1H] (7.21), 219 (100), 91 (9.40), 64 (6.25); Anal. for C_22_H_17_ClN_2_O_3_ (392.83) calcd; C, 67.26; H, 4.36; Cl, 9.02; N, 7.13; Found: C, 67.13; H, 4.35; Cl, 9.08; N, 7.14.

*2-(4-Chlorophenyl)-3-(3,4-dimethoxyphenethyl)quinazolin-4(3H)-one* (**3c**). **Compound** **3c** was prepared from 2-amino-*N*-(3,4-dimethoxyphenethyl)benzamide **1c** according to method A, beige cubes; yield (78**^a^**%); m.p. 150 °C; IR *ν*_max_ (KBr) 1681.50, 1614.63 cm^−1^; ^1^H-NMR (400 MHz, DMSO-*d*_6_) (ppm): *δ* 7.65 (1H, d, *J* = 8.0 Hz), 7.34–7.44 (5H, m), 7.20 (1H, t, *J* = 8.0 Hz), 6.83 (1H, d, *J* = 8.4 Hz), 6.75 (1H, s), 6.68 (1H, t, *J* = 8.0 Hz), 6.63 (1H, d, *J* = 8.4 Hz), 3.70, 3072 (each 3H, s, 2(OCH_3_)), 2.96 (2H, t, *J* = 6.9 Hz, CH_2_), 2.65 (2H, t*,J* = 7.3 Hz, CH_2_); ^13^C-NMR: *δ* 20.0, 35.0, 47.5, 55.8, 112.39, 112.97 (2C), 114.82,115.43, 117.85, 121.03 (2C), 128.00, 128.73 (2C), 129.09 (2C), 131.91, 133.55, 133.86, 140.62, 146.73, 147.80, 149.10, 162.7; MS *m*/*z* (%): 168[M^+^](C_24_H_21_N_2_O_3_Cl); Anal. for C_24_H_21_ClN_2_O_3_ (420.89) calcd; C, 68.49; H, 5.03; Cl, 8.42; N, 6.66; Found: C, 68.51; H, 5.02; Cl, 8.40; N, 6.68.

*2-(4-(2-(4-Chlorophenyl)-4-oxoquinazolin-3(4H)-yl)phenyl)acetic Acid* (**3d**). **Compound** **3d** was prepared from 2-(4-(2-aminobenzamido)phenyl)acetic acid **1d** according to method A, shining beige needles; yield (65**^a^**%); m.p. 213 °C; IR *ν*_max_ (KBr) 3653.46, 3109.55, 1707.22, 1665.45, 1620.90 cm^−1^; ^1^H-NMR (400 MHz, DMSO-*d*_6_) (ppm): *δ* 12.32 (1H, s, OH), 8.00 (1H, d, *J* = 7.5 Hz), 7.71(1H, t, *J* = 7.5 Hz), 7.60–7.66 (4H, m), 7.42 (1H, d, *J* = 7.5 Hz), 7.33 (2H, d, *J* = 7.3 Hz), 7.28 (1H, t, *J* = 7.5 Hz), 7.21 (2H, d, *J* = 7.3 Hz), 3.34 (2H, s, CH_2_); ^13^C-NMR: *δ* 20.0, 115.43, 115.87, 120.06, 126.47(2C), 128.97(2C), 129.77, 130.00(2C), 131.17, 133.17(2C), 133.47,134.43, 139.70, 140.33, 146.86, 162.70, 173.1, 173.4; MS *m*/*z* (%): 390[M^+^] (C_22_H_15_N_2_O_3_Cl) (3.12), 389 [M^+^−C_22_H_15_ClN_2_O_3_ (390.82) calcd; C, 67.61; H, 3.87; Cl, 9.07; N, 7.17; Found: C, 67.60; H, 3.90; Cl, 9.10; N, 7.18.

### 3.3. Antimicrobial Evaluation [30]

#### 3.3.1. Antifungal Activity

Samples of the synthesized molecules were subjected separately *in vitro* for their antifungal evaluation viz. *Aspergillus fumigatus* (RCMB 002003), *Geotrichum candidum* (RCMB 052006) *Candida albicans* (RCMB 005002) and *Syncephalastrum racemosum* (RCMB 005003). The cluture of fungi was purified by single spore isolation technique. The antifungal activity was tested by agar well diffusion method according to the following procedure:

Sabourad dextrose agar plates: A homogeneous mixture of glucose-peptone-agar (40:10:15) was sterilized by autoclaving at 121 °C for 20 min. The sterilized solution (25 mL) was poured in each sterilized petri dish in a laminar flow hood and left for 20 min. to form the solidified sabourad dextrose agar plate. These plates were inverted and kept at 30 °C in an incubator to remove the moisture and to check for the contamination.

#### 3.3.2. Antifungal Assay

Fungal strain was grown in 5 mL sabourad dextrose broth (glucose/peptone; 40:10) for 3–4 days to achieve 105C FU/mL cells. The fungal culture (0.1 mL) was spread out uniformly on the sabourad dextrose agar plates by sterilized triangular folded glass rod. Plates were left for 5–10 min. so that culture is properly adsorbed on the surface of sabourad dextrose agar plates. Small wells of size (4 mm × 2 mm) were cut into the plates with the help of well cutter and bottom of the wells were sealed with 0.8% soft agar to prevent the flow of test sample at the bottom of the well. 100 μL of the tested samples (10 mg/mL) were loaded into the wells of the plates. All compounds dissolved in DMSO were loaded as control. The plates were kept for incubation at 30 °C for 3–4 days and then the plates were examined for the formation of zone of inhibition. Each inhibition zone was measured three times by caliper to get an average value. The test was performed three times for each fungus. Clotrimaole and itraconazole were used as antifungal standard drugs.

#### 3.3.3. Antibacterial Activity

Antibacterial evalution was tested using agar well diffusion method. The activity of tested samples was tested against *Staphylococcus aureus* (RCMB 000106) and *Bacillis subtilis* (RCMB 000107), as Gram positive bacteria and *Pseudomonas aeruginosa* (RCMB 000102) and *Escherichia coli* (RCMB 000103), as gram negative bacteria. The solution of 5 mg/mL of each compound in DMSO was used for testing against bacteria. Centrifuged pellets of bacteria from 24 h old culture containing approximately 104–106 CFU (colony forming unit) per mL were spread on the surface of nutrient agar (typetone 1%, yeast extract 0.5%, NaCl 0.5%, agar 1000 mL of distilled water, pH 7.0) and was autoclaved under 121 °C for at least 20 min. Wells were created in medium with the help of sterile metallic bores and then cooled down to 45 °C. The activity was determined by measuring the diameter of the inhibition zone (in mm). 100 μL of the tested samples (10 mg/mL) were loaded into the wells of the plates. All compounds were prepared in DMSO, and were loaded as control. The plates were kept for incubation at 37 °C for 24 h and then the plates were examined for the formation of zone of inhibition. Each inhibition zone was measured three times by caliper to get an average value. The test was performed three times for each bacteria. Streptomycin was used as an antibacterial standard drug.

#### 3.3.4. Material and Methods

To investigate the binding pattern of the Quinazoline derivatives respect to their binding affinity, docking experiments were performed using genetic algorithm approach implemented in the GOLD [33] docking program for conformational search during docking process. PDB ID 1T2W retrieved from Protein Data Bank [34] and the co-crystallized ligand LPXTG peptide complex along with water moelcules were omitted from the targeted protein. Default parameters were set to optimize the docking experiment. All the Ligands were sketched by ChemDraw Ultra and converted into 3D format using OpenEye Babel [35]. Hydrogen’s were added and MMFF94 partial charges were assigned to all ligands by MOE [36] prior to minimization. Furthermore, protonation states were corrected using OpenEye Filter [37] program before docking runs. MOE and Poseview [38] were used for the molecular interaction analysis.

## 4. Conclusions

We have successfully prepared quinazolin-4(3H)-one derivatives **2a**–**h** and **3a**–**d** starting from 2-aminobenzamide derivatives **1** with triethyl orthoformate and *p*-chlorobenzaldehyde, respectively, by one pot synthesis assisted by microwave or classical methods. The simple procedure, mild conditions, high yields and especially environmental friendliness make this protocol very attractive. Compound **3a** showed strong inhibitory effect against Gram-negative bacteria (*Pseudomonas aeruginosa* and *Esherichia coli*) and Gram-positive bacteria (*Staphylococcus aureus*, and *Bacillus subtilis*), in addition to and anti-fungal activity against (*Aspergillus fumigatus*, *Saccharomyces cervevisiae*, and *Candida albicans*).
